# Optimized reusable modular 3D-printed models of choledochal cyst to simulate laparoscopic and robotic bilioenteric anastomosis

**DOI:** 10.1038/s41598-024-59351-6

**Published:** 2024-04-16

**Authors:** Jing Gu, Jie Cao, Wenli Cao, Yusuo Chen, Fangqiang Wei

**Affiliations:** 1 Department of General Surgery, Cancer Center, Division of Hepatobiliary and Pancreatic Surgery, Zhejiang Provincial People’s Hospital, Affiliated People’s Hospital, Hangzhou Medical College, Hangzhou, 310014 Zhejiang Province China; 2 Second Clinical Medical College, Zhejiang Chinese Medical University, Hangzhou, 310053 Zhejiang Province China; 3 Ningbo Chuangdao 3D Medical Technology Co., Ltd., Ningbo, 315336 Zhejiang Province China; 4 Department of Public Health, Hangzhou Medical College, Hangzhou, 310059 Zhejiang Province China; 5 Department of Clinical Medicine, Hangzhou Medical College, Hangzhou, 310059 Zhejiang Province China

**Keywords:** 3D-printed model, Laparoscopic, Robotic, Bilioenteric anastomosis, Training, Anatomy, Gastroenterology, Medical research

## Abstract

Laparoscopic and robotic surgery is a challenge to the surgeon's hand–eye coordination ability, which requires constant practice. Traditional mentor training is gradually shifting to simulation training based on various models. Laparoscopic and robotic bilioenteric anastomosis is an important and difficult operation in hepatobiliary surgery. We constructed and optimized the reusable modular 3D-printed models of choledochal cyst. The aim of this study was to verify the ability of this optimized model to distinguish between surgeons with different levels of proficiency and the benefits of repeated practice. A total of 12 surgeons with different levels participated in the study. Operation completion time and OSATS score were recorded. The model was validated by Likert scale. Surgeons were shown the steps and contents before performing laparoscopic or robotic bilioenteric anastomosis using the model. Surgeons with different levels of experience showed different levels when performing laparoscopic bilioenteric anastomosis on this model. Repeated training can significantly shorten the time of laparoscopic bilioenteric anastomosis and improve the operation scores of surgeons with different levels of experience. At the same time, preliminary results have shown that the performance of surgeons on the domestic robotic platform was basically consistent with their laparoscopic skills. This model may distinguish surgeons with different levels of experience and may improve surgical skills through repeated practice. It is worth noting that in order to draw more reliable conclusions, more subjects should be collected and more experiments should be done in the future.

## Introduction

Compared with traditional open surgery, laparoscopic surgery has unique advantages such as less trauma, clearer surgical vision, shorter hospital stays, less blood loss, and faster postoperative recovery^1–3^. Safety has also been verified in malignancies such as liver cancer, gastric cancer, and pancreatic cancer, and the long-term prognosis of patients is similar to that of open surgery^4–6^. With the continuous improvement of laparoscopic-related instruments and surgeons' operating techniques, the scope of application of laparoscopic surgery is also expanding. Similarly, robotic surgery is gaining increasing popularity among patients, with similar or even better perioperative outcomes compared to laparoscopic surgery^7^. Nowadays, laparoscopic surgery and robotic surgery have become the surgical treatment of choice for many diseases. While laparoscopic surgery and robotic surgery can be trained with more practice, testing hand–eye coordination remains difficult. Notably, there is still a certain threshold in practice, and unskilled operation may result in damage to surrounding tissues and inaccurate suture, which will affect the occurrence of serious postoperative after surgery^8^. With the improvement of patients’ requirements for medical quality, the requirements for surgeons will inevitably increase. Based on the above points, this requires surgeons to receive more training in laparoscopic surgery and robotic surgery. At present, there are many forms of training, such as traditional training, video training, wet laboratory training, dry laboratory training^9–12^, etc. With the maturity of 3D printing technology, dry laboratory training based on 3D printing models provides a safe, effective and cost-effective training environment outside the operating room^12^.

Bilioenteric anastomosis is a relatively complex surgical procedure in hepatobiliary surgery, which requires high technical requirements for the surgeon. It is widely used in surgeries requiring biliary duct reconstruction, such as choledochal cyst resection, radical resection of hilar cholangiocarcinoma, and pancreaticoduodenectomy. Unskilled operation may lead to serious postoperative complications^13^. At present, there are few reports on simulation training of laparoscopic bilioenteric anastomosis^14–16^, and there is a lack of mature and reliable training model. There have been no reports of training using 3D-printed models of choledochal cysts that can be reused for laparoscopic or robotic bilioenteric anastomosis. Therefore, we constructed and optimized our previously reported reusable modular 3D-printed models^17^, focusing on choledochal cysts. The aim of this study was to verify the ability of this optimized model to distinguish between surgeons with different levels of proficiency and the benefits of repeated practice of bilioenteric anastomosis.

## Methods

### Constructing 3D-printed models

**Figure 1:**
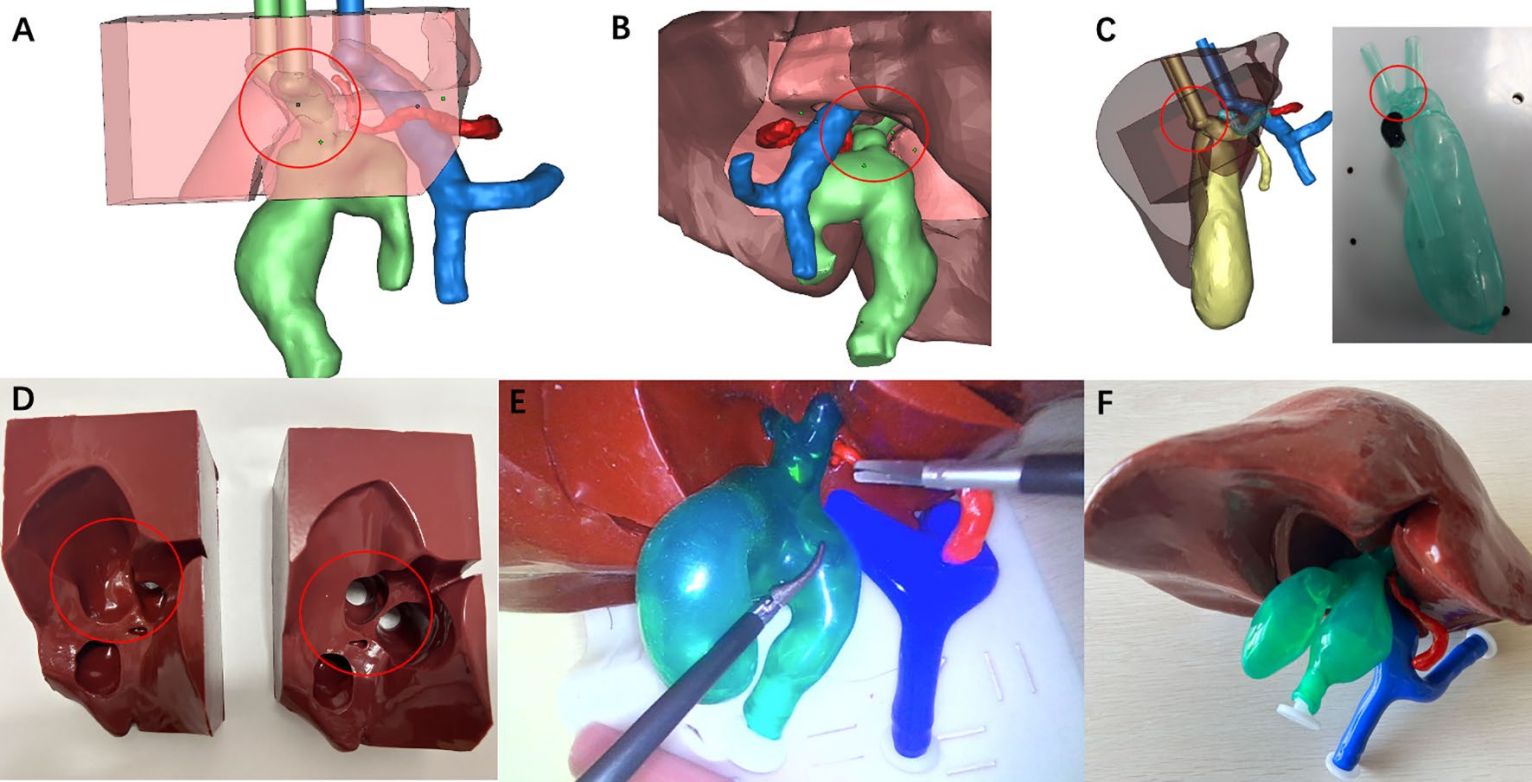
Optimized reusable modular 3D-printed models of choledochal cyst. (**A**, **B**) Electronic design of replacement blocks. The red circle displays junction between the left and right hepatic duct and the common hepatic duct. (**C**) Electronic design and real model of our previously reported model of extrahepatic cholangiocarcinoma. The red circle displays the junction between the left and right hepatic duct and the common hepatic duct. (**D**) The real model of the replacement blocks. On the left is the optimized replacement block, and on the right is the original replacement block. The circles represent the grooves in the replacement block that connect to the bile duct structure. (**E**, **F**) Model presentation of optimized reusable modular 3D-printed models of choledochal cyst.

**Figure 2:**
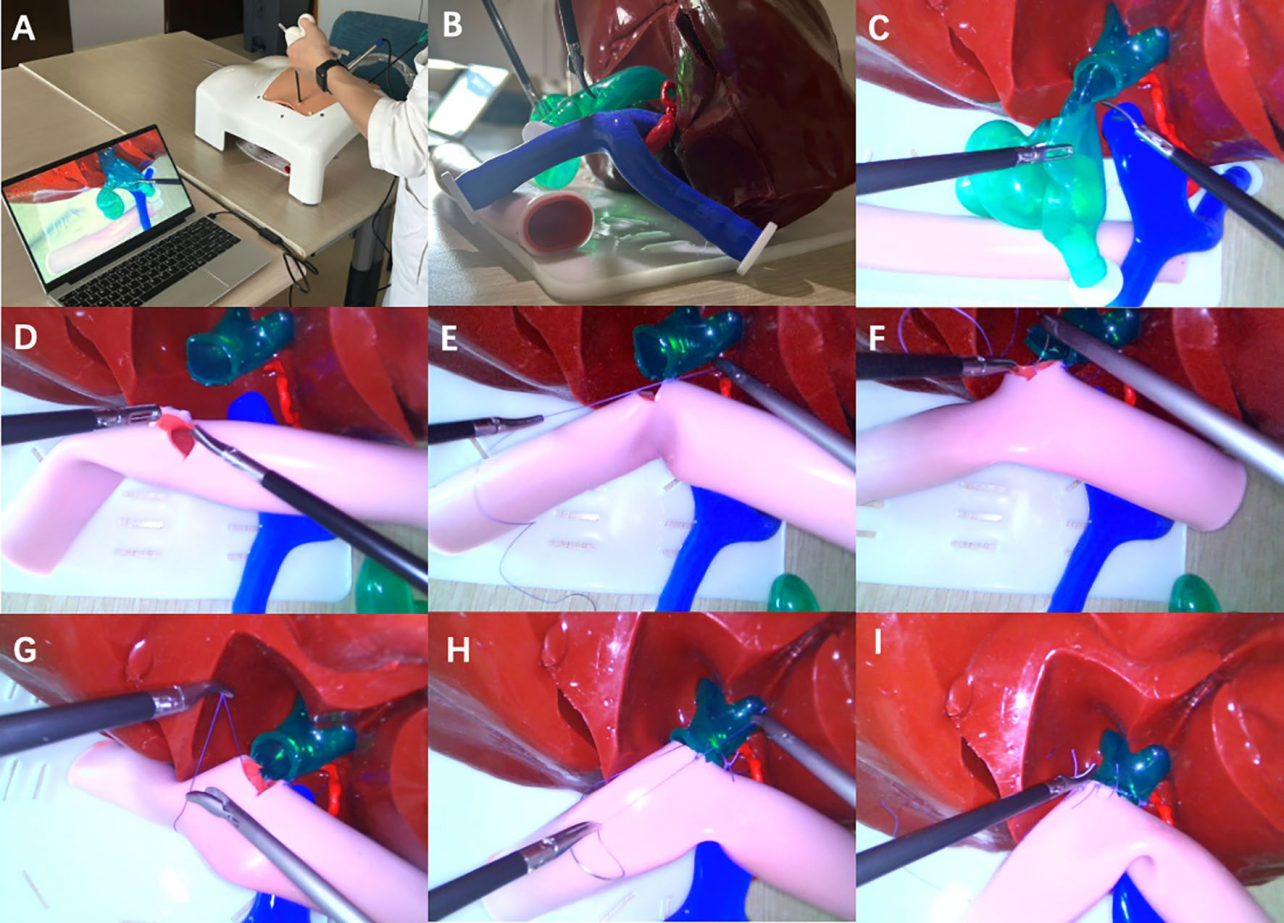
Laparoscopic bilioenteric anastomosis training based on the 3D-printed models. (**A**) Overall diagram of the operation. (**B**) Model presentation. (**C**) Choledochal cyst resection. (**D**) Preparing for the anastomosis. (**E**–**G**) Anastomosis of the posterior wall of the common hepatic duct. (**H**–**I**) Anastomosis of the anterior wall of the common hepatic duct.

**Figure 3:**
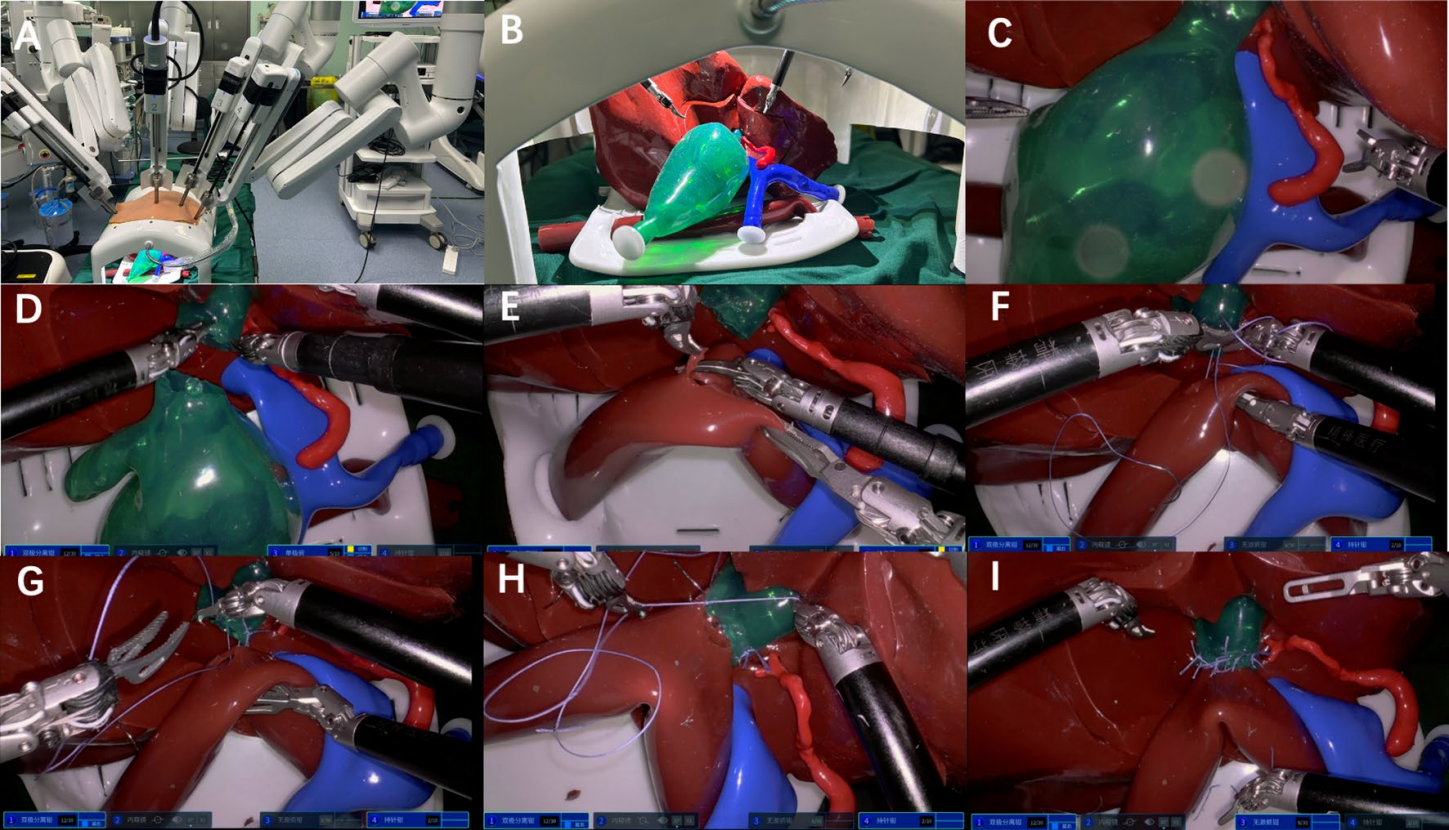
Robotic bilioenteric anastomosis training based on the 3D-printed models. (**A**) Overall diagram of the operation. (**B**, **C**) Model presentation. (**D**) Choledochal cyst resection. (**E**) Preparing for the anastomosis. (**F**, **G**) Anastomosis of the posterior wall of the common hepatic duct. (**H**, **I**) Anastomosis of the anterior wall of the common hepatic duct.

We have constructed and optimized our previously reported reusable modular 3D-printed models^17^ (Fig. 1). Anonymized Digital Imaging and Communication in Medicine (DICOM) files were achieved from CT or MRI scans of human choledochal cyst lesions, including two adult males and two adult females (weighed 52, 61, 58, and 43 kg, respectively). All the 4 reconstructed models of choledochal cyst were Todani type I. The maximum diameters of choledochal cysts in the four models were 2.8 cm (Fig. 1A,B,E), 4 cm (Fig. 1F), 4 cm (Fig. 2), and 5 cm (Fig. 3), respectively. In order to allow participants to experience the realistic simulation of choledochal cyst resection and ensure the training effect of each bilioenteric anastomosis, the diameter of the common hepatic duct was maintained constant at 1.5 cm (with a thickness of 1.5 mm) in each reconstructed choledochal cyst model. Multiple copies of the model were printed out and randomly assigned to participants. The first practice of this model can remove a choledochal cyst. Due to the constant diameter of the common hepatic duct, three subsequent times of bilioenteric anastomosis were relatively fixed.

Importantly, we optimized the model by replacing the replacement block so that it could assemble a different choledochal cyst model (Fig. 1). Specifically, the previous model was an extrahepatic cholangiocarcinoma model^17^, and the junction between the left and right hepatic duct and the common hepatic duct is relatively short and straight (Fig. 1C), which is compatible with the corresponding replacement block grooves. The optimized choledochal cyst model is long and curved at the junction of the left and right hepatic duct and the common hepatic duct, and the grooves of the replacement block have been optimized (Fig. 1D). After optimization, the groove design of the replacement block can make the choledochal cyst model more adaptable and stretchable, so as to avoid the high position of the bilioenteric anastomosis and the difficulty of bilioenteric anastomosis. In addition, due to the optimization of the replacement blocks and the fixed diameter of the common hepatic duct, different types of choledochal cyst models can be well reassembled to meet the needs of repetitive training.

Regarding the related software, the 3D reconstruction and design were completed using the E-3D Digital Medical Modeling and Design System V19.12 (Digital Health and Virtual Reality Research Center, Central South University, China). Slicing was performed using the open source slicing software Cura 4.4.1 (Ulitmaker, USA) for 3D printing. The reusable modular 3D-printed models were produced by double extruded silicone 3D printer (Silplot-S400, Ningbo Chuangdao 3D Medical Technology Co., LTD., China). Besides, a jejunum model (made of silicon, 23 mm diameter with a length of 150 mm and a thickness of 2.5 mm) (Fig. 2), together with the abdominal simulator (the length, width and height are 40 cm, 32 cm and 20 cm, respectively) (Fig. 2A), both of which are commercially purchased (ExtrainSurge Medical Technology Co., LTD., Hangzhou, China) are available for laparoscopic and robotic bilioenteric bilioenteric anastomosis. As mentioned, the diameter of the common hepatic duct, which is available for bilioenteric anastomosis, in each reconstructed choledochal cyst model is constant, with a diameter of 1.5 cm and a thickness of 1.5 mm.

### Training program

#### Study participants

Table 1 presents the details of participants. A total of 12 surgeons participated in the training. All the 12 participants were from our institution and all volunteered to participate in the study. They were invited to fill out a basic information form after they voluntarily agreed to participate in the study. All the 12 participants were males, and they were divided into three groups according to their age, working years, and surgical experience. Specifically, there were 4 resident surgeons (group A, average age 26.3 ± 1.5 years, average working years 1.75 ± 0.5); 4 senior surgeons (group B, average age 33.0 ± 1.6 years, average working years 4.3 ± 0.9); 4 expert surgeons (group C, average age 40.5 ± 5.6 years, average working years 12.5 ± 5.1). Four residents were from the general surgery standardized training program, and all four residents had first assistant experience in more than 50 cases of laparoscopic cholecystectomy, but had never performed the surgery alone. Four senior surgeons and four expert surgeons had individual experience of performing of laparoscopic cholecystectomy (average cases 152.5 ± 119.8 for senior surgeons, 1037.5 ± 110.9 for expert surgeons) and laparoscopic common bile duct exploration (average cases 13.8 ± 13.6 for senior surgeons, 267.5 ± 101.1 for expert surgeons). All the 12 participants were right-handed.

**Table 1:** General characteristics.

	Group A (n = 4)	Group B (n = 4)	Group C (n = 4)	P value A vs B	P value C vs B
Age (years)	26.3 ± 1.5	33.0 ± 1.6	40.5 ± 5.6	0.001	0.041
Gender (male/female)	4/0	4/0	4/0	NS	NS
Working year	1.75 ± 0.5 (Duration of training)	4.3 ± 0.9	12.5 ± 5.1	0.004	0.019
Number of cases of achieved surgeries					
LC	0	152.5 ± 119.8	1037.5 ± 110.9	0.044	< 0.001
LCBDE	0	13.8 ± 13.6	267.5 ± 101.1	NS	0.002

Group A: Resident surgeon; Group B: Senior surgeon; Group C: Expert surgeon.

LC: laparoscopic cholecystectomy; LCBDE: laparoscopic common bile duct exploration. NS: Not significant.

#### Laparoscopic equipment and materials

A 30° camera (Lap Game, Jingyou Technology Co., LTD, Hangzhou, China) is inserted into the simulated umbilical site of the abdominal simulator as observation (equivalent to a No. 2 arm in robotic surgery), which is linked to a regular laptop with Windows 64-bit operating system as the monitor through the software of YouCam5 (CyberLink, China). Two surgical ports are located about 12 cm outside the left and right sides of the umbilical level, respectively (in robotic surgery, the left side is No. 1 arm and the right side is No. 4 arm) (Figs. 2A, 3A). During laparoscopic surgical suture, the separation forceps (Lap Game, Jingyou Technology Co., LTD, Hangzhou, China) is placed in the left and the needle driver (Lap Game, Jingyou Technology Co., LTD, Hangzhou, China) is placed in the right. Suturing was performed using a 26 mm-diameter 1/2 curved needle with the purple 3-0 VICRYL absorbable suture material (VCP774D, Ethicon, Inc. Texas, USA).

#### Robotic equipment and materials

In robotic surgery, the domestic robotic platform called Jingfeng porous surgical robot (MP1000, Shenzhen Edge Medical Co., Ltd, China) is used. There are four arms, each of which is fitted with a 10 mm diameter port. The No.2 arm places the camera (Image processor, MP1202; Monitor, SONY LMD-X2705MC) used as observation. Except another two operating arms (No. 1 and No. 4 arms) mentioned above, an additional No. 3 arm (placing the grasping forceps, MP1305) is also located between the No. 1 arm (placing the separation forceps, MP1302) and the No. 4 arm (placing the needle driver, MP1308). The port layout has been just described as the above, and the materials used were similar as the above.

#### Surgical steps

Surgeons were shown the steps and contents before performing procedures using the model. The specific operation steps were as follows. First, the choledochal cyst was removed and a small incision was made in the intestine. Second, the intestine was lifted to the side of the common hepatic duct, and the posterior wall of the common hepatic duct was sutured to the intestine continuously, while the anterior wall of the common hepatic duct was sutured to the intestine intermittently or continuously. Repeat the second steps two more times. In addition, three surgeons were invited to complete the simulation training of robotic bilioenteric anastomosis based on the domestic robotic platform (Shenzhen Edge Medical Co., Ltd, China).

#### Assessment

The optimized model was validated from five aspects including the impression, realism, esthetics, usability, and handling, utilizing the 5-grade Likert scale (1, 2, 3, 4, 5 represents, strongly disagree, disagree; neither agree nor disagree; agree; strongly agree; respectively). Specifically, this evaluation included five aspects as follows: (1) Impression. The model is generally considered helpful for surgical training; (2) Realism. The model is similar to a real organ or tissue. (3) Esthetics. The model is considered to meet esthetic standards; (4) Usability. The model is easy to use; (5) Handling. The model can simulate surgical operations. The performance of surgeons was evaluated by modified Objective Structured Assessment of Technical Skills (OSATS) score (Table S1). The OSATS scores were analyzed by two experts familiar with OSATS scores by reviewing the recorded videos and scoring them independently and anonymously. These two experts are surgical specialists in hepatobiliary and pancreatic surgery from our institution. The completion time of each simulated operation was also calculated.

### Data analysis

All statistical analyses were performed with the use of SPSS, version 26.0 (IBM Corp.). Quantitative data are presented as mean ± standard deviation. Independent sample t test was utilized to compare the two groups. Count data were compared by Fisher exact test. A P value less than 0.05 was defined statistically significant.

### Ethics approval and consent to participate statement

The current study was approved by the committee of Zhejiang Provincial People's Hospital. The study was conducted in accordance with the Declaration of Helsinki. The study gathered data from surgeons’ training based on 3D printed models and all surgeons gave informed consent.

## Result

### General characteristic

A total of 12 surgeons participated in the training. All of the 12 surgeons were male, including 4 resident surgeons (group A, average age 26.3 ± 1.5 years); 4 senior surgeons (group B, average age 33.0 ± 1.6 years); 4 expert surgeons (group C, average age 40.5 ± 5.6 years). The longer the working years, the richer the surgical experience achieved among the three groups (Table 1**)**.

### Model evaluation

The model was evaluated by surgeons from five aspects including the impression, realism, esthetics, usability, and handling, all of which were highly recognized. There was no significant difference between the groups (Table 2**)**.

**Table 2:** Model scoring.

Model scoring	Group A (n = 4)	Group B (n = 4)	Group C (n = 4)	P value A vs B	P value C vs B
Impression	4.8 ± 0.5	4.8 ± 0.5	4.8 ± 0.5	NS	NS
Realism	4.8 ± 0.5	4.5 ± 0.6	4.3 ± 0.5	NS	NS
Esthetics	4.5 ± 0.6	4.8 ± 0.5	4.8 ± 0.5	NS	NS
Usability	4.8 ± 0.5	4.8 ± 0.5	4.5 ± 0.6	NS	NS
Handling	4.3 ± 0.5	4.5 ± 0.6	4.5 ± 0.6	NS	NS

Group A: Resident surgeon; Group B: Senior surgeon; Group C: Expert surgeon.

NS: Not significant.

### Operation assessment

All surgeons performed the operation successfully (Fig. 2). The OSATS score significantly improved after training and the completion time significantly decreased when the training was progressing (Tables 3 and 4). In the comparison of surgeons with different experience, the more qualified surgeons performed better than the less qualified surgeons (Tables 3 and 4).

**Table 3:** Operation score.

Groups	Completion time (min)	OSATS
Group A		
First time (A1)	59.3 ± 7.9	2.1 ± 0.3
Second time (A2)	47.0 ± 10.4	2.6 ± 0.5
Third time (A3)	34.8 ± 7.6	2.8 ± 0.1
Group B		
First time (B1)	38.8 ± 7.4	3.3 ± 0.2
Second time (B2)	28.3 ± 2.4	3.5 ± 0.1
Third time (B3)	24.5 ± 3.7	3.8 ± 0.1
Group C		
First time (C1)	27.5 ± 7.5	4.3 ± 0.2
Second time (C2)	19.5 ± 4.3	4.5 ± 0.3
Third time (C3)	17.6 ± 3.9	4.6 ± 0.1

Group A: Resident surgeon; Group B: Senior surgeon; Group C: Expert surgeon.

Data are presented as mean ± standard deviation.

**Table 4:** Comparison of operation scores.

Groups	Completion time, P value	OSATS, P value
A1A3	0.004	0.002
B1B3	0.014	0.007
C1C3	0.029	0.045
A1B1	0.009	< 0.001
B1C1	0.049	< 0.001
A1C1	0.001	< 0.001

### Robotic bilioenteric anastomosis simulation training

Three surgeons, from group A(Aa), B(Bb), C(Cc) with different levels of experience were invited to complete robotic simulation training of bilioenteric anastomosis (Fig. 3). The completion time and OSATS scores of achieving robotic bilioenteric anastomosis was 65 min, 56 min and 21 min, and 2.8, 3.3 and 4.5, respectively, for surgeon A(Aa), B(Bb), C(Cc), which were basically consistent with their laparoscopic performance (Tables 3 and 5).

**Table 5:** Training scores using domestic robotics.

Participants	Completion time (min)	OSATS
Aa		
First	65	2.8
Bb		
First	56	3.3
Cc		
First	21	4.5

## Discussion

Currently, the surgical teaching practice model has gradually changed from the traditional master-apprentice model to multi-mode surgical training models^9,10,12,18,19^. Laparoscopic surgery simulation training methods mainly include video training, wet laboratory training, dry laboratory training, and virtual reality training^10–12,19^. Although watching the operation video is mainly to improve the anatomical knowledge of beginners, it does not translate into the improved surgical skills^10^. If you watch the operation technique video, you may teach yourself the laparoscopic suture knotting technique^20^. Wet laboratory training relies on cadaveric animals or animal models, such as sheep^11^ or ex vivo pig liver^21^ as training models for laparoscopic hepatectomy. Although such animal models offer a more realistic experience, they have limitations such as cost, anesthesia, ethical approval, and are not portable and difficult to preserve. Smagister is a new platform that combines live animal models with ex vivo simulation^22^. Although it provides an extremely realistic simulation environment, its high cost, including equipment rental fees, the purchase of ex vivo tissues and organs and preservation solutions, as well as a short storage time, more suitable for high-level laparoscopic surgery training, is still too extravagant for the novice. Dry laboratory training relies on synthetic models, currently mainly 3D-printed silicone or hydrogel models^12,15,23^, which are easy to carry, reusable, do not require animal models, and do not involve issues such as ethical approval. Virtual reality training is the focus of current research. The latest systematic review^19^ recognizes its training effectiveness, but there is no significant difference in the learning curve compared to other training methods. Moreover, due to the lack of validation of skill transfer from trainees to operating room, virtual reality training is not suitable as a first choice for laparoscopic training. However, it can stimulate the interest of inexperienced medical or novice surgeons to participate in laparoscopic surgery and help improve the learning curve in the initial stages. According to the above training types, for laparoscopic surgery training, different training methods at different stages may achieve the best training results. The training method based on 3D printing model is the most widely used laparoscopic training method after comprehensive consideration, which is also more conducive to the understanding of anatomical knowledge for beginners^24^. Based on the above situation, we constructed and optimized our previously reported reusable modular 3D-printed models^17^. Herein focusing on choledochal cysts, this paper provides surgeons, with different proficiency levels, with as realistic a simulation scene as possible to repeatedly practice bilioenteric anastomosis.

For the completion time of the first laparoscopic bilioenteric anastomosis, experienced surgeons operate for a shorter time, so resident surgeons operate for longer (59.3 ± 7.9 vs. 38.8 ± 7.4 min) than senior surgeons, resident surgeons operate for longer (59.3 ± 7.9 vs. 27.5 ± 7.5 min) than expert surgeons, and senior surgeons operate for longer (38.8 ± 7.4 vs. 27.5 ± 7.5 min) than expert surgeons (all P < 0.05); Similarly, experienced surgeons performed better on OSATS scores. After three times of simulation practice, the completion time of laparoscopic bilioenteric anastomosis was shortened in all groups (all P < 0.05), with resident surgeons 59.3 ± 7.9 vs. 34.8 ± 7.6 min, senior surgeons 38.8 ± 7.4 vs. 24.5 ± 3.7 min, and expert surgeons 27.5 ± 7.5 vs. 17.6 ± 3.9 min. Similarly, OSATS scores increased in all groups (all P < 0.05), with resident surgeons 2.1 ± 0.3 vs. 2.8 ± 0.1, senior surgeons 3.3 ± 0.2 vs. 3.8 ± 0.1, and expert surgeons 4.3 ± 0.2 vs. 4.6 ± 0.1. The improvement was even greater for surgeons with less surgical experience, especially for resident surgeons (completion time 59.3 ± 7.9 vs. 34.8 ± 7.6 min, OSATS scores 2.1 ± 0.3 vs. 2.8 ± 0.1, both p < 0.05). Even expert surgeons showed small but significant improvements in their simulation performance after three practice sessions (completion time 27.5 ± 7.5 vs. 17.6 ± 3.9 min, OSATS scores 4.3 ± 0.2 vs. 4.6 ± 0.1, both p < 0.05). The above results have shown that this training model has the ability to distinguish surgeons of different levels and improve the surgical level of surgeons of different levels through repeated training. At the same time, we also carried out simulation training on the domestic robotic platform. Although the young surgeons who participated in the training had less experience with simulated robotic surgery, their OSATS scores were basically consistent with their laparoscopic surgery training. This suggests that there may be a correlation between extensive experience in laparoscopic techniques and performance on robotic platforms. The robotic platform provides a three-dimensional view and a more flexible and intuitive operation field, which to a certain extent also helps surgeons to complete the operation better^25^. In the future, with the large-scale procurement of domestic robotic platforms in China, surgeons with rich experience in laparoscopic surgery will be able to better apply domestic robotic platforms.

The modular choledochal cyst model we constructed was rated highly on all five dimensions of the model score, giving basically similar and relatively high ratings on impression and esthetics. The results have shown that optimized reusable modular 3D-printed models has a good appearance and may provide surgeons with a relatively realistic simulated surgical environment. Our models have the following advantages. First, the modular model design allows us to replace choledochal cyst models and reduce the cost. Second, after the completion of the first bilioenteric anastomosis, the anastomotic bile duct could be cut off, repeated training could be used, and the low to high bilioenteric anastomosis could be simulated, gradually increasing the difficulty of operation and further balancing the cost of each use. Third, models based on 3D printing technology can be extended to be used for preoperative surgical planning and simulated surgical practice training^26^. Fourth, unlike traditional organs such as pig liver, it is easy to carry and preserve, and there are no animal ethical problems.

At the same time, the model score also reflects some shortcomings of the model. In the model evaluation, although there was no statistically significant difference, the more experienced surgeons gave lower realism scores. Experienced surgeons are more likely to detect the difference between the silicone models and real organs. On the other hand, models may not be as useful for junior surgeons as they are for senior surgeons. For junior surgeons who have just received the residency training, due to direct contact with simulated bilioenteric anastomosis model training, lack of early transition practice, it is difficult to adapt to the first time, resulting in difficulty in completion of surgery, long completion time, low operation score, giving a relatively low handling score. This may affect junior surgeons’ motivation to learn.

This study has the following limitations. First, limited by the number of models and consumables required to simulate surgery, the sample size of the included subjects was small, and the number of repeated training was small. Especially for robotic anastomosis, data from only three surgeons doing it once is scientifically insufficient. Although the results are preliminary, we found that the model performed well on both laparoscopic and robotic platforms. However, with the increase of training resources and opportunities, more subjects should be collected and more times should be done in the future to confirm our hypotheses with a more solid conclusion. Second, due to the small number of female surgeons available and the lack of female surgeons in the study, there is a lack of data on the training of female surgeons in this study. Third, the simulated surgical environment established by the 3D model is relatively simple, including only the liver, portal vein, hepatic artery, bile duct, without surrounding structures such as the pancreas, omentum and intestines as in the real environment. Moreover, there are differences in the texture and other factors between the model and the actual environment, which raises the question of whether operations in the simulated environment can effectively simulate operations in the real operating room. Fourth, there is a lack of real laparoscopic or robotic bilioenteric anastomosis data in the operating room environment after simulation training, so it is impossible to evaluate whether the training effect is trully translated into actual effect.

## Conclusion

Our model is currently one of the few 3D-printed models that can simulate laparoscopic or robotic bilioenteric anastomosis. This model may distinguish surgeons with different levels of experience and may improve surgical skills through repeated practice. Therefore, we believe that this model can be used to further improve the quality of laparoscopic training courses for residents, and also to evaluate the level of surgical experience of surgeons at all levels. It is worth noting that in order to draw more reliable conclusions, more subjects should be collected and more experiments should be done in the future.

### Supplementary Information

Supplementary Information.

## Data Availability

The data gathered in the current study are available from the corresponding author on reasonable request.
