# Supplementary material for: Optimized reusable modular 3D-printed models of choledochal cyst to simulate laparoscopic and robotic bilioenteric anastomosis
Source: Sci Rep. 2024 Apr 16;14:8807. doi: 10.1038/s41598-024-59351-6 (PMC11021543; doi:10.1038/s41598-024-59351-6)
Supplement: Supplementary file 1 — Supplementary Information. [file 41598_2024_59351_MOESM1_ESM.docx]

**Table S1.** Modified Objective Structured Assessment of Technical Skills ^1,2^

| **Gentleness** | | | | |
| --- | --- | --- | --- | --- |
| 1 | 2 | 3 | 4 | 5 |
| Frequently used unnecessary force on tissues or caused damage by inappropriate use of instruments |  | Careful handling of tissues but occasionally caused inadvertent damage |  | Consistently handled tissues appropriately with minimal damage |
| **Time and Motion** | | | | |
| 1 | 2 | 3 | 4 | 5 |
| Many unnecessary moves |  | Efficient time/motion but some unnecessary moves |  | Clear economy of movement and maximum efficiency |
| **Instrument Handling** | | | | |
| 1 | 2 | 3 | 4 | 5 |
| Repeatedly makes tentative or awkward moves with instruments by inappropriate use of instruments |  | Competent use of instruments but occasionally appeared stiff or awkward |  | Fluid moves with instruments and no awkwardness |
| **Flow of Operation** | | | | |
| 1 | 2 | 3 | 4 | 5 |
| Frequently stopped operating and seemed unsure of next move |  | Demonstrated some forward planning with reasonable progression of procedure |  | Obviously planned course of operation with effortless flow from one move to the next |
| **Tissue exposure** | | | | |
| 1 | 2 | 3 | 4 | 5 |
| Poorly or failed to expose operative field |  | Appropriate exposure of operative field |  | Always expose the best operative field |
| **Overall technical skill** | | | | |
| 1 | 2 | 3 | 4 | 5 |
| Deficient |  | Average |  | Proficient |

1、Martin JA, Regehr G, Reznick R, et al. Objective structured assessment of technical skill (OSATS) for surgical residents. Br J Surg. 1997;84:273-278.

2、Birkmeyer JD, Finks JF, O'Reilly A, et al. Surgical skill and complication rates after bariatric surgery. N Engl J Med. 2013;369:1434-42.
